# Safety of Receiving Anti–Vascular Endothelial Growth Factor Intravitreal Injection in Office-Based vs Operating Room Settings: A Meta-analysis

**DOI:** 10.1001/jamaophthalmol.2021.3096

**Published:** 2021-08-19

**Authors:** Tong Li, Junran Sun, Jingyu Min, Shuangwen Zhou, Xiaolin Zhu, Huixun Jia, Xiaodong Sun

**Affiliations:** 1 Department of Ophthalmology, Shanghai General Hospital, Shanghai Jiao Tong University School of Medicine, Shanghai, China; 2 National Clinical Research Center for Ophthalmic Diseases, Shanghai, China; 3 Novartis Pharmaceuticals, Shanghai, China; 4 Shanghai Engineering Center for Visual Science and Photomedicine, Shanghai, China; 5 Shanghai Key Laboratory of Fundus Diseases, Shanghai, China

## Abstract

**Importance:**

Compared with the operating room (OR), office-based intravitreal injection (IVI) is considered a more cost-effective and convenient approach, yet clinical outcomes of IVIs with anti–vascular endothelial growth factor (VEGF) agents in different settings (office-based vs OR) have not been systematically evaluated.

**Objective:**

To evaluate the safety outcomes of IVI with anti-VEGF agents in the OR vs office-based setting.

**Data Sources:**

PubMed, Embase, Cochrane Library, Web of Science, and ClinicalTrials.gov were searched from inception to July 2020.

**Study Selection:**

Eligible studies reporting on patients who received IVIs with anti-VEGF drugs with a clearly stated injection setting of the office or OR.

**Data Extraction and Synthesis:**

Two reviewers independently screened studies, extracted data, and assessed risk of bias. A meta-analysis was conducted to determine the rates of endophthalmitis (EO) and culture-positive EO.

**Main Outcomes and Measures:**

Rates of EO and culture-positive EO following anti-VEGF IVIs in the OR and office-based setting.

**Results:**

Thirty-one studies with a total of 1 275 815 injections were included. Comparative analysis suggested no difference between rates of EO after IVIs performed in the office and OR settings (odds ratio, 3.06; 95% CI, 0.07-139.75; *P* = .57; *I*^2^ = 80%) were identified, yet a higher rate of culture-positive EO was found in the office setting (odds ratio, 21.52; 95% CI, 2.39-193.55; *P* = .006; *I*^2^ = 0%). The pooled rates of EO following anti-VEGF IVIs were 0.03% (95% CI, 0.03-0.04) and 0.02% (95% CI, 0.01-0.04) in office and OR settings, respectively, and the pooled rates of culture-positive EO were 0.01% (95% CI, 0.01-0.02) and 0.01% (95% CI, 0-0.02). The pooled rates of other ocular and systemic adverse events were low.

**Conclusions and Relevance:**

The rate of clinically suspected or culture-positive EO following anti-VEGF IVIs was low whether the procedure was performed in the office or OR setting. Bacterial spectrum could differ between the 2 settings. This meta-analysis could not determine if it is more appropriate to give treatment in the OR for safety reasons in low-income compared with higher-income regions in the world.

## Key Points

**Question:**

Are the safety outcomes of intravitreal injection with anti–vascular endothelial growth factor agents in the office-based setting comparable with outcomes in the operating room?

**Findings:**

In this meta-analysis including 31 studies with a total of 1 275 815 injections, no difference between rates of postinjection endophthalmitis were identified in the 2 settings. The rates of other ocular adverse events were quite low, with no systemic adverse events reported.

**Meaning:**

This study cannot determine whether performing injection in the operating room is more appropriate in lower-income regions.

## Teaser

This meta-analysis evaluates the safety outcomes of intravitreal injection with anti–vascular endothelial growth factor agents in the operating room vs office-based setting.

## Introduction

First described at the turn of the 20th century for repairing retinal detachments (RDs), intravitreal injections (IVIs) have become the most widely performed procedures in the ophthalmic field.^1^ IVIs with anti–vascular endothelial growth factor (VEGF) agents have significantly improved management and visual prognosis of angiogenic retinal diseases, including neovascular age-related macular degeneration, diabetic macular edema, retinal vein occlusion, proliferative diabetic retinopathy, choroidal neovascularization, and other visual impairments.^2^

Endophthalmitis (EO) is a severe form of eye inflammation that may result in irreversible blindness if the intraocular infection is not properly treated.^3^ To our knowledge, the largest meta-analysis on EO to date revealed an overall rate of 0.06% (197 of 350 535 injections) following IVIs with all anti-VEGF agents.^4^ Anti-VEGF injections require repeated injections on a regular basis.^5^ Despite that subsequent injections did not increase the risk of EO compared with the first one, awareness of the risk remains clinically relevant as the number of injections increases.^4^

IVIs with anti-VEGF agents are predominantly performed in the operating room (OR) in Europe and in resource-limited countries, such as China^6^ and India,^7^ owing to the perception of a decreased risk of infection.^8^ Given the high volume of IVIs performed annually with limited availability of ORs, these injections were typically administered in an office setting in the US.^9,10,11^ Iatrogenic infection remains a primary concern for office-based IVI,^12^ yet evidence from primary research remains controversial; while Abell et al^13^ found a significantly higher risk of EO following office-based IVIs than in the OR (relative risk, 13.1; 95% CI, 1.5-112.4), no significant difference was observed between the 2 settings in the study by Tabandeh et al^8^ (odds ratio, 0.53; 95% CI, 0.09-3.18). These findings suggest a need for systemic evidence on EO rates and other safety outcomes following anti-VEGF IVIs in the 2 settings. Here, we conducted a meta-analysis evaluating the safety of anti-VEGF IVI in the office and OR settings and discuss the implications for future practice.

## Methods

This study was performed based on the Meta-analysis of Observational Studies in Epidemiology (MOOSE) reporting guideline.^14^

### Literature Search Strategies

PubMed, Embase, Cochrane Library, Web of Science, and ClinicalTrials.gov were searched from database inception to July 2020. The literature search was restricted to English-language articles using a combination of subject wording, keywords, and free-text terms, including *Lucentis*, *ranibizumab*, *Eylea*, *aflibercept*, *conbercept*, *Beovu*, *brolucizumab*, *bevacizumab*, *anti-VEGF*, *operating room*, *office-based*, *intravitreal injection*, and *IVI*. Details of the search are shown in the eMethods in the Supplement. Bibliographic searches of relevant reviews, guidelines, and meta-analysis were conducted to identify additional studies.

### Eligibility Criteria

A study was eligible for inclusion if the following criteria were met: (1) the study focused on patients who received IVI with anti-VEGF drugs alone or combined with panretinal photocoagulation, laser, or photodynamic therapy; (2) there was a clearly stated injection setting of office or OR; (3) the article reported the safety outcomes following anti-VEGF IVIs; and (4) the study was primary clinical research, including randomized clinical trials (RCTs), nonrandomized comparative cohorts, cross-sectional studies, and case series.

### Study Selection

Two reviewers (T.L. and J.S.) independently screened the titles and abstracts to assess the initial eligibility of a study. Full texts of initially eligible studies were retrieved for detailed inspection. Discrepancies between the 2 reviewers were resolved through discussion and consultation with a senior reviewer (H.J.) when necessary. Studies that were not omitted after full-text screening were considered suitable for inclusion.

### Data Extraction and Quality Assessment

Variables extracted included (1) study characteristics (first author, publication year, study period, geographic location, number of patients/eyes, age, sex, dominant indication, study design, and follow-up duration), (2) intervention and/or comparison (anti-VEGF drug, injection settings, injection number, same-day bilateral injection, sterile condition, and drug preparation and doses), and (3) details of a specific safety outcome. The methodological quality was evaluated according to the design of each study included: RCTs were evaluated using the revised Cochrane risk-of-bias tool (https://www.riskofbias.info), case series were assessed with the National Institute for Clinical Excellence criteria,^15^ and case-control and comparative cohort studies were assessed with the Newcastle-Ottawa Scale.^16^

### Outcome Measures

Safety outcomes of this study included rates of EO, culture-positive EO, RD, vitreous hemorrhage, intraocular pressure elevation, intraocular inflammation, and other ocular and systemic adverse events (AEs). Each outcome was summarized according to the settings.

### Statistical Analysis

Meta-analysis was performed when 2 or more studies reported the same outcome; otherwise, results were separately described for any study with a different definition of outcomes. Quantitative data analysis was conducted using R software version 4.0.2, META package (The R Foundation). Peto odds ratio method was used for analysis of dichotomous variables when event rates were less than 1%; otherwise, Mantel-Haenszel method was used.^17^ Considering that the risk of EO might be associated with number of injections, rates of EO were combined with number of EO out of the total number of injections, which is in line with previous studies.^10,18^ Generalized linear mixed models with the logit link were used for pooled analysis of single proportions to account for within-study uncertainties.^19,20^

Heterogeneity across the studies was examined using *I*^2^ statistics. The estimates were pooled with a fixed-effect model when no significant heterogeneity (*I*^2^ less than 50%) was observed; otherwise, a random-effect model was used. Sensitivity analysis was performed evaluating the impact of country income level on injection safety.^21^ Publication bias was assessed by Egger test for any outcome with 10 or more studies included. *Q* test was used to calculate *P* values for heterogeneity, while *z* test was used for overall effect. Two-sided *P* values less than .05 were considered statistically significant.

## Results

### Study Selection and Characteristics

**Figure 1.**
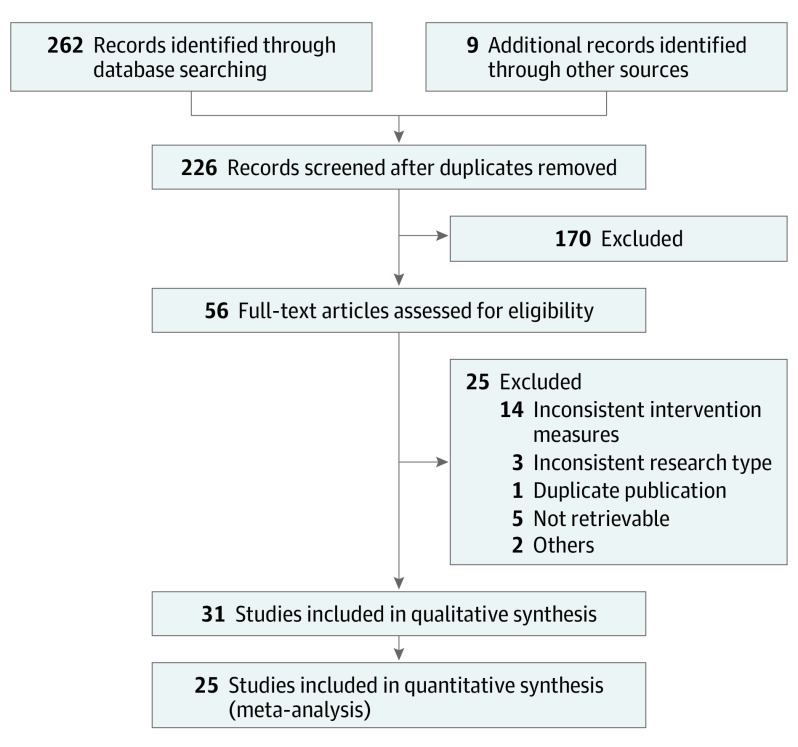
Study Selection Process

Figure 1 shows the selection process of studies identified through the database search, with 262 initial records and 9 additional records through other sources. Ultimately, 31 studies with a total of 1 275 815 injections were included for qualitative synthesis.

The baseline characteristics of the included studies are detailed in eTable 1 in the Supplement. Seventeen studies reported a total of 944 765 injections administered in the office setting,^8,13,22,23,24,25,26,27,28,29,30,31,32,33,34,35,36^ while 14 studies reported 302 039 injections administered in the OR.^6,7,8,13,37,38,39,40,41,42,43,44,45,46^ Only 2 studies directly compared office-based injections with the OR-based injections: Abell et al^13^ reported no significant difference in age, sex, ocular comorbidities, or socioeconomic status between the 2 injection settings, while Tabandeh et al^8^ indicated similar baseline diagnosis; patients in both studies were injected with ranibizumab or bevacizumab. A total of 28 studies (90%) reported low risk of bias in 50% or more items in methodological quality assessment (eTable 2 in the Supplement). Prophylaxis measures for postinjection infection are shown in eTable 3 in the Supplement.

### Overall Rates of EO

**Figure 2.**
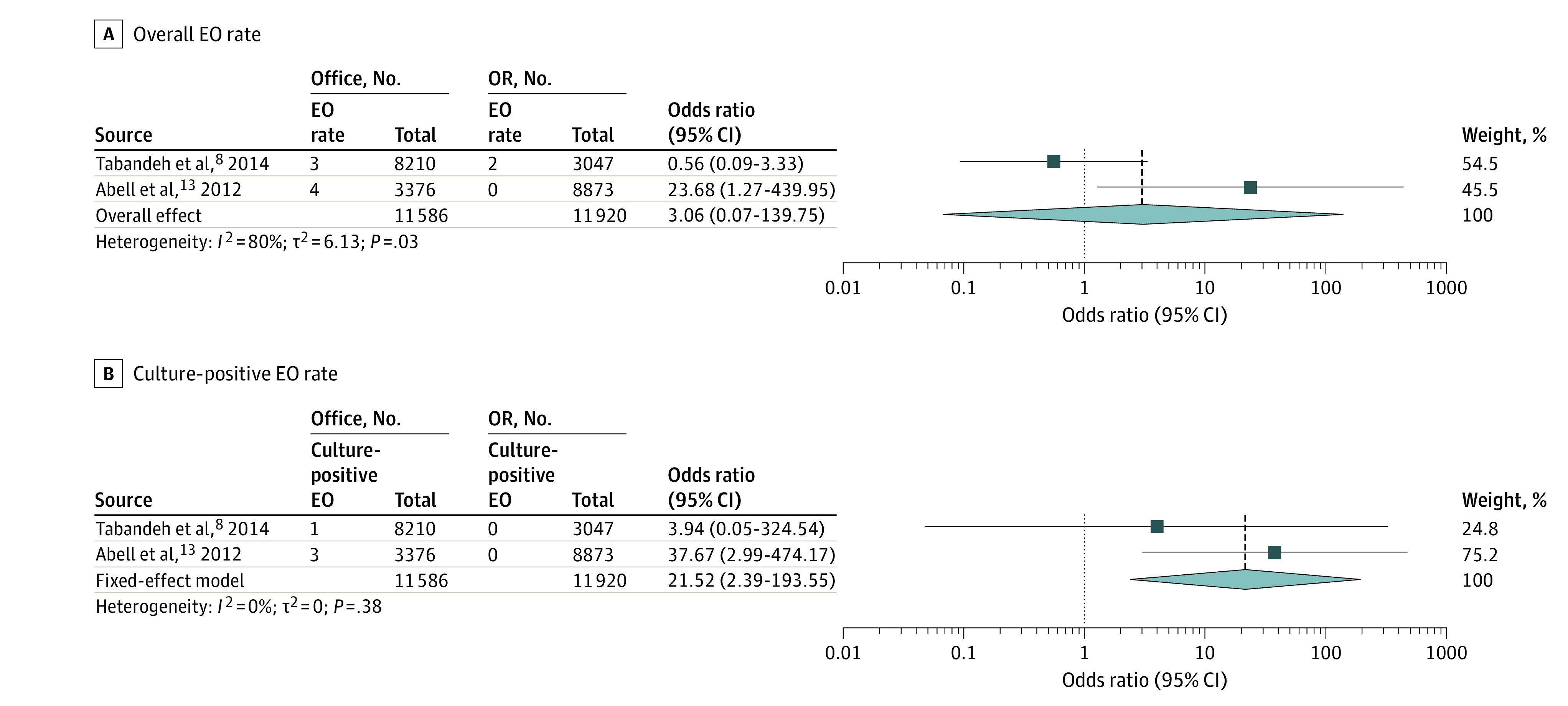
Forest Plot of Overall Endophthalmitis (EO) Rate and Culture-Positive EO Rate in the Office vs the Operating Room (OR) Setting

**Figure 3.**
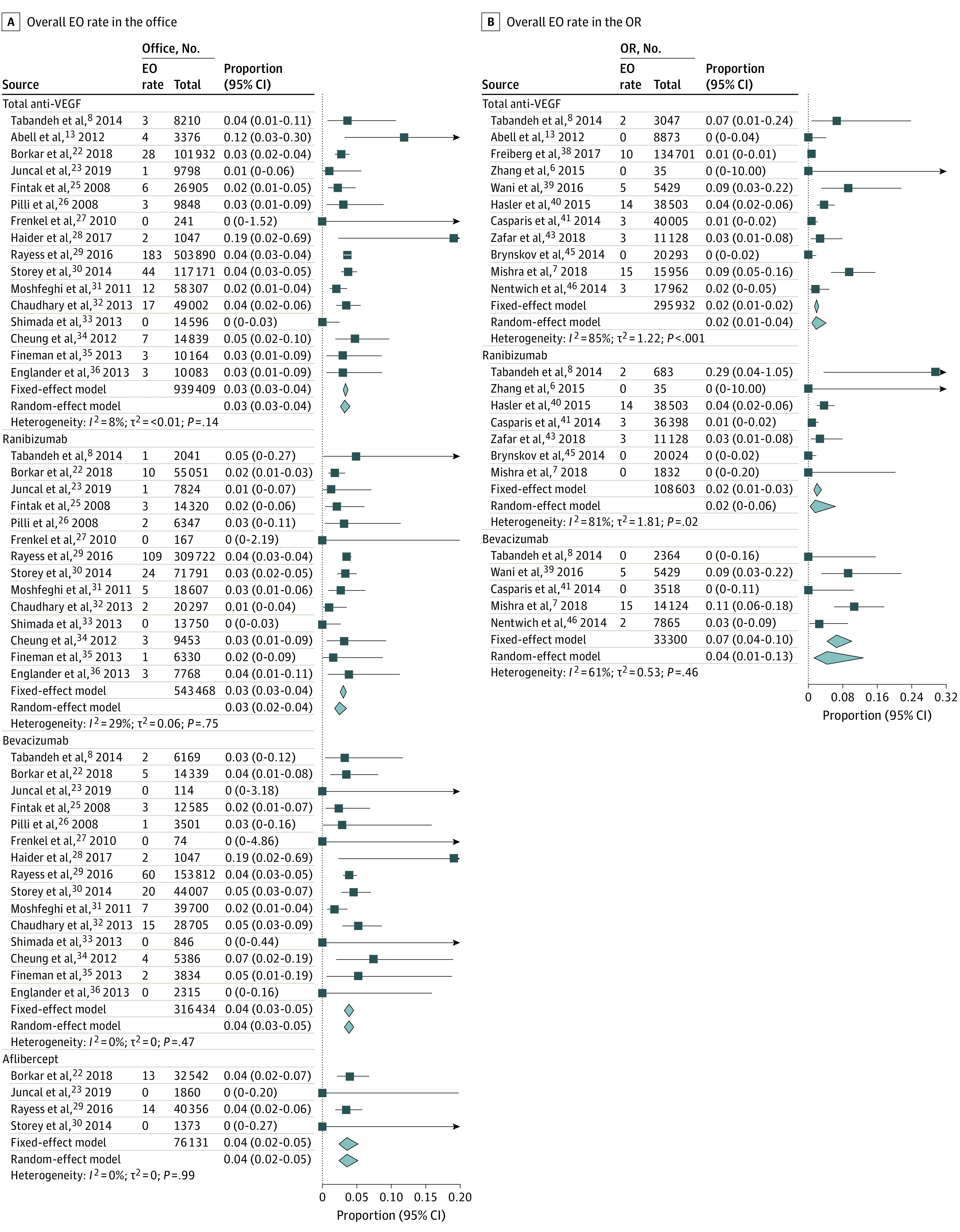
Forest Plot of Pooled Rate of Overall Endophthalmitis (EO) Following Intravitreal Injection in the Office vs Operating Room (OR) Setting

For comparative analysis (Figure 2A),^8,13^ there was no difference between the 2 injection settings (odds ratio, 3.06; 95% CI, 0.07-139.75; *P* = .57), but significant heterogeneity was observed (*I*^2^ = 80%). The pooled rates of EO in the office and OR settings are presented in Figure 3.^6,7,8,13,22,23,25,26,27,28,29,30,31,32,33,34,35,36,38,39,40,41,43,45,46^ In the office setting, the rates of EO following IVI of total anti-VEGF drugs, ranibizumab, bevacizumab, and aflibercept were 0.03% (95% CI, 0.03-0.04), 0.03% (95% CI, 0.03-0.04), 0.04% (95% CI, 0.03-0.05), and 0.04% (95% CI, 0.02-0.05), respectively. In the OR setting, the rates of EO following IVI of total anti-VEGF drugs, ranibizumab, and bevacizumab were 0.02% (95% CI, 0.01-0.04), 0.02% (95% CI, 0-0.06), and 0.04% (95% CI, 0.01-0.13), respectively. There was no significant heterogeneity for total anti-VEGF drugs, ranibizumab, bevacizumab, and aflibercept in the office setting (*I*^2^ = 8%; *I*^2^ = 29%; *I*^2^ = 0%; *I*^2^ = 0%, respectively), while statistical heterogeneity was found for all 3 groups in the OR setting (*I*^2^ = 85%; *I*^2^ = 81%; *I*^2^ = 61%). Additionally, 2 studies reported no EO following 358 IVIs of aflibercept in the OR.^41,45^

### Rates of Culture-Positive EO

**Figure 4.**
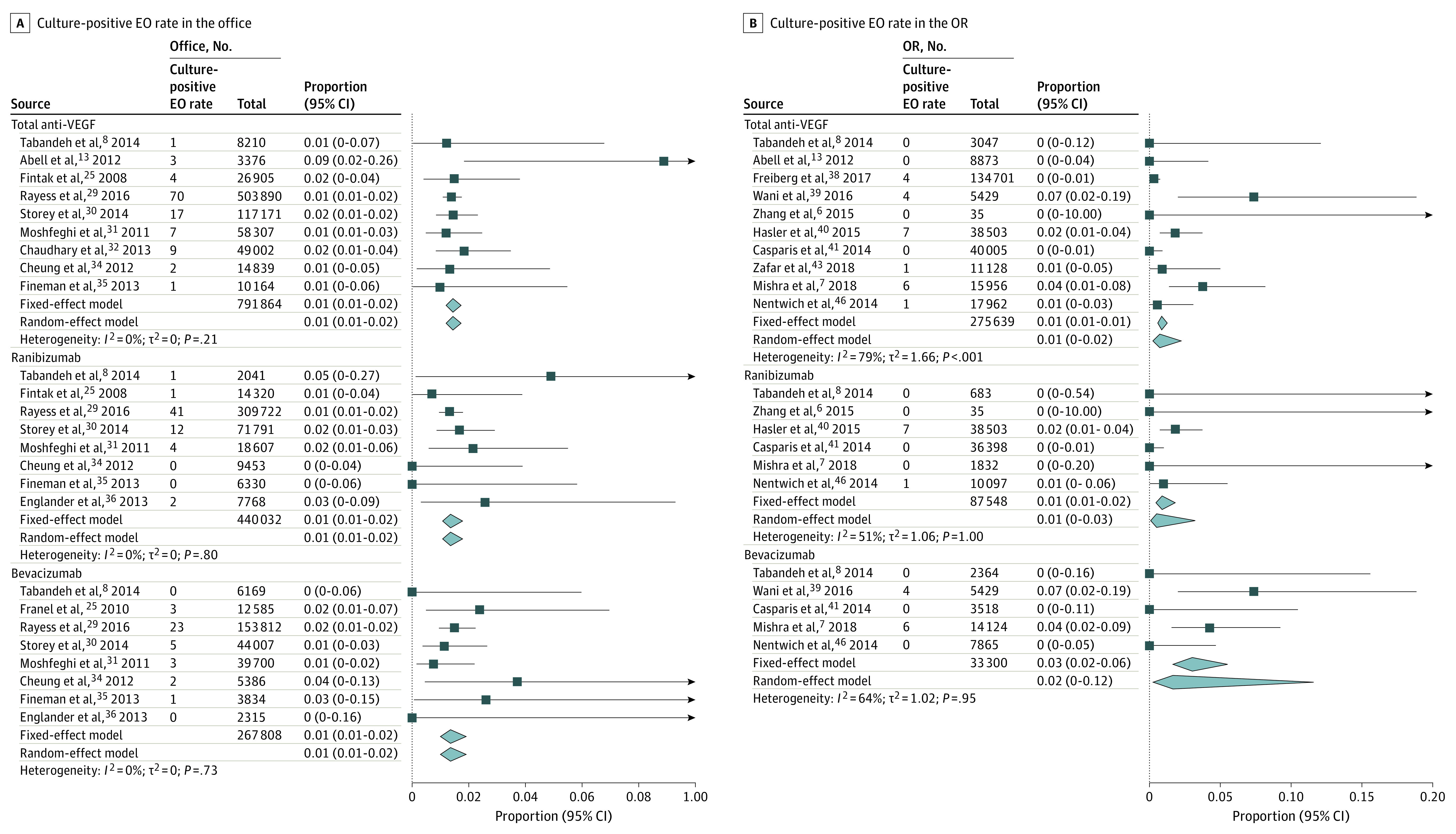
Forest Plot of Pooled Rate of Culture-Positive Endophthalmitis (EO) Following Intravitreal Injection in the Office vs Operating Room (OR) Setting

The culture-positive EO rate was significantly higher in the office setting compared with the OR setting, with no statistical heterogeneity being observed (odds ratio, 21.52; 95% CI, 2.39-193.55; *P* = .006; *I*^2^ = 0%) (Figure 2B). The pooled rates of culture-positive EO in the office and OR settings are presented in Figure 4.^6,7,8,13,25,27,29,30,31,32,34,35,36,38,39,40,41,43,46^ The rates of culture-positive EO following IVI of total anti-VEGF drugs, ranibizumab, and bevacizumab were 0.01% (95% CI, 0.01-0.02), 0.01% (95% CI, 0.01-0.02), and 0.01% (95% CI, 0.01-0.02), respectively, in the office setting with no statistical heterogeneity (*I*^2^ = 0%; *I*^2^ = 0%; *I*^2^ = 0%). In the OR setting, rates of culture-positive EO following IVI of total anti-VEGF drugs, ranibizumab, and bevacizumab were 0.01% (95% CI, 0-0.02), 0.01% (95% CI, 0-0.03), and 0.02% (95% CI, 0-0.12), respectively, with significant heterogeneity (I^2^ = 79%; *I*^2^ = 51%; *I*^2^ = 64%).

### Microbiologic Spectrum of Culture-Positive EO

**Figure 5.**
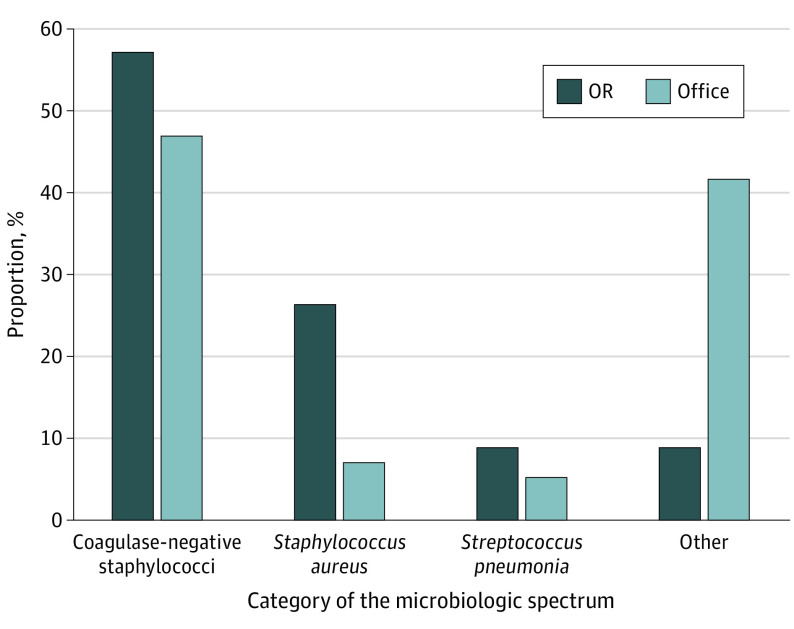
Microbiologic Spectrum of Culture-Positive Endophthalmitis Following Intravitreal Anti–Vascular Endothelial Growth Factor Injection in the Office vs Operating Room (OR) Setting Others in the office setting include *Streptococcus viridans*, *Haemophilus, Enterococcus, Bacillus*, *Candida*, and *Actinobacteria*. Others in the OR setting include group D* streptococci* and *Proteus *species.

Sixteen studies reported a total of 11 microbial species accounting for the EO (Figure 5).^7,8,13,25,29,30,31,32,34,35,36,38,39,40,43,46^ In the office setting, the 2 most common pathogens were Coagulase-negative staphylococci (CoNS) and *Streptococcus viridans* (46.5% [53 of 114] and 25.4% [29 of 114], respectively), whereas in the OR, the predominant pathogens were CoNS and *Staphylococcus aureus* (57% [13 of 23] and 26% [6 of 23], respectively).

### Sensitivity Analysis

The only study in the office setting of a resource-limited country had the highest EO rate across all office-based studies.^28^ Removing this study did not significantly alter the overall effect and heterogeneity. For studies in the OR, EO rates were overall higher in resource-limited countries than higher-income ones (eFigures 1 to 5 in the Supplement).

### Other Ocular and Systemic AEs

No patient developed RD according to 2 studies^23,27^ in the office setting and 1 study^6^ in the OR, whereas another study^40^ reported an RD rate of 0.003% in the OR. Two studies in the office setting^23,27^ gave a combined incidence rate of 0.01% and 0.02% for vitreous hemorrhage and RT, respectively. One case of cataract (0.003%) occurred in the OR,^40^ but none occurred in the office setting.^27^ Twenty cases of noninfectious inflammation were reported following 5356 aflibercept injections administered in 7 office locations in the US.^24^ In total, 5 studies,^6,23,27,42,44^ including 2 in the office and 3 in the OR, claimed no systemic complications.

### Publication Bias

No publication bias was indicated for outcomes with 10 or more studies included (eFigure 6 in the Supplement).

## Discussion

IVIs of anti-VEGF agents are conducted in the OR because of the perception that the negative pressure environment of the OR can effectively reduce the risk of intraocular infections.^38,47^ However, a growing number of practitioners in high-income countries, such as the US, UK, Japan, Australia, and Canada, began to perform office-based IVIs as it is a more cost-effective, convenient, and efficient approach without compromising safety.^10,11,13,23,24,25,33,34,47,48,49^ In these 25 studies, we found an overall EO rate of 0.03% (95% CI, 0.03-0.04) in the office setting and 0.02% (95% CI, 0.01-0.04) in the OR. McCannel^18^ reported an overall EO rate of 0.05% (95% CI, 0.04-0.07) following office-based anti-VEGF IVIs. Sigford et al^11^ used EO rates in the US (0.05%) and Europe (0.03%) as surrogates to the estimated rates in the office and OR settings, respectively. Through pooling the studies with a well-described setting, our results were in general comparable with the previous reports.

Our analysis revealed a culture-positive EO rate of 0.01% (95% CI, 0.01-0.02) in the office setting and 0.01% (95% CI, 0-0.02) in the OR setting. Our comparative analysis, however, observed that culture-positive EO rates were significantly elevated in the office setting, yet this finding may be overestimated, as socioeconomic status was a potential confounder of the injection setting in the study by Abell et al.^13^ In line with previous studies,^18,50^ CoNS accounted for most culture-positive EO cases in both settings. As a normal flora typically found on the skin and conjunctiva, the spread of CoNS during the injection procedure (that is, from a patient’s conjunctiva and lacrimal apparatus to the vitreous body) is a notable cause of infectious EO.^51^ Almost all studies included in our analysis reported povidone-iodine use prior to administering an IVI. Application of topical povidone-iodine for at least 30 seconds before each IVI can effectively reduce conjunctival bacteria counts and has become the standard of care for prophylaxis of intraocular infection.^9,47,52^ *S viridans* was predominantly isolated in the office settings, which is consistent with findings from Busch et al^2^ that a decreased rate of Streptococcus-induced EO was reported after IVIs in the OR. *S viridans* is a normal bacterial flora typically found in the oral cavity, upper respiratory tract, and gastrointestinal tract.^51^ It is postulated that talking or coughing during the IVI procedure, especially in the office setting where a face mask is not a standard of care, increases the chance of needle or conjunctiva contamination.^13,50,51^ Most of our studies in the OR reported using sterile gloves, masks, speculum, drape, and hand antisepsis, but many studies in the office setting either did not report or reported nonadequate use of 1 or more of these aseptic techniques. As the vitreous is an immune-privileged site susceptible to infection even under a small inoculum of low virulence bacteria, adherence to masking and silence and other aseptic measures throughout the procedure are warranted to decrease bacterial dispersion.^18,53,54^

Young age^55^ and female sex^55,56,57^ have been identified as risk factors associated with EO after IVIs of anti-VEGF medications. However, more than two-thirds of the included studies did not report patients’ age or sex (eTable 1 in the Supplement), thus restricting us from further analyzing their impact within each injection setting (OR vs office). In a retrospective cohort of 818 558 IVIs, Kiss et al^4^ found a more than 2-fold elevated risk of EO associated with aflibercept than ranibizumab, yet our pooled EO rates were not different between aflibercept, ranibizumab, and bevacizumab. Sterile inflammation has been reported in patients receiving aflibercept IVIs.^24,58^ Since noninfectious and infectious EO may share similar symptoms, such as pain, conjunctival infection, and hypopyon, differential diagnosis can be difficult and at the discretion of the treating ophthalmologist.^29,58^ Therefore, different diagnostic criteria may account for the various EO rates reported after aflibercept injection.

Overall and culture-positive EO rates for ranibizumab appear higher in the office setting, while rates for bevacizumab seem higher in the OR, yet none of these results were statistically meaningful. Our findings give the implication that not only a clean environment with strict aseptic rules during the injection procedure but also the degree to which sterile techniques were used for syringe preparation may also affect postinjection EO. Ranibizumab, initially available as single-dose vials, received its first prefilled syringe approval by the European Union in 2013 and the US Food and Drug Administration in 2016 for 0.5 mg and 0.3 mg specifications.^59^ Through simplifying the process of drawing up medication from vials, use of prefilled syringe was shown to effectively reduce the risk of contamination and subsequent postinjection EO regardless of the anti-VEGF medication inside.^56,57^ Ophthalmic use of bevacizumab was in the scope of off-label application; as preparation requires fractioning from the original vial into multiple injections, bacterial burden may accumulate by multiple punctures of the rubber cap of the vial.^7,39,60^ In the US, bevacizumab syringes are commonly prepared by compounding pharmacies that have to comply with the US Pharmacopeia.^26,56^ Bevacizumab was repackaged at compounding pharmacies in all of our studies in the office setting with available information on drug preparation, whereas 3 of 5 studies in the OR reported multiple withdrawals from a single vial by the treating physician. The relatively higher EO rates after bevacizumab administration in the OR may be a result of suboptimal aseptic rules followed during drug preparation in a setting other than the standard compounding pharmacy.^12^ Owning to the sterile drug prepackaging procedures in a controlled environment, Bavinger et al^56^ found that among 1 095 305 IVIs, compared with prefilled bevacizumab syringes at compounding pharmacies, office-filled ranibizumab and aflibercept together increased postinjection EO (odds ratio, 1.29; *P* = .02).

From our analysis and previous studies,^61^ injection setting did not appear to be an influential factor of postinjection EO. In a study of 14 001 anti-VEGF IVIs delivered in a procedure room dedicated for IVI only and with strict aseptic rules, serious ocular AEs, including EO, traumatic cataract, and RD, were observed only in 1 (0.01%), 3 (0.02%), and 1 (0.01%) cases, respectively.^62^ Therefore, we propose that a well-established IVI protocol together with sterile environment and aseptic procedure preparation are key to the safe delivery of anti-VEGF IVIs. Based on our sensitivity analysis in the OR setting, overall and culture-positive EO rates together with that of the bevacizumab subgroup were significantly higher in resource-limited countries than higher-income ones, yet no difference was found in the ranibizumab subgroup (eFigures 1 to 5 in the Supplement). However, we cannot determine from the data analyzed whether office-based injections in low-income regions have any greater rate of EO. The fact that there is a strong regional difference in setting, especially among OR-based studies where a significant heterogeneity of the data are observed, is a large limiting factor in making accurate comparisons.

The pooled rates of other ocular and systematic AEs were rare (1 or more per 10 000 to less than 1 per 1000) or very rare (less than 1 per 10 000) in both settings. Incorrect injection technique is a primary cause of posterior vitreous detachment; our findings were consistent with the previous report of a low rate (0 to 0.67%) of RD and RT following anti-VEGF IVI.^63^ In a meta-analysis of 8 RCTs evaluating the systemic safety of intravitreal anti-VEGF agents for treatment of retinal vein occlusion, anti-VEGF drugs did not increase the risks of cardiovascular events compared with placebo/retinal photocoagulation.^10^

### Limitations

There are several limitations to our study. First, given that EO rates are very low, findings may not be conclusive based on the current sample size. Second, information bias is possible, as the included studies almost exclusively focused on EO; other safety outcomes were rarely reported. Third, most studies are retrospective case series, which provides lower levels of evidence than RCTs and prospective cohorts. However, as the rates of EO were very low, prospective trials are not feasible and may lack clinical relevance even in the presence of statistical significance.^47^ Fourth, the significant heterogeneity of data from OR-based injections may limit the strength of our findings. Fifth, since only 2 studies are directly comparative, our analysis was largely restricted to indirect comparison.

## Conclusions

In conclusion, the rate of clinically suspected or culture-positive EO following anti-VEGF IVIs was low whether the procedure was performed in the office or OR. Bacterial spectrum can differ between the 2 settings. In resource-limited regions, we were unable to identify evidence that in-office procedures would lead to more EO than injections in the OR. Using antisepsis from drug packaging and loading the syringes to the completion of IVIs in a well-controlled, clean environment may contribute to prophylaxis of postinjection EO.

## Notes

Supplement. **eMethods.** Search strategies. **eTable 1.** Baseline characteristics of the included studies. **eTable 2.** Methodological qualities of the included studies. **eTable 3.** Characteristics of the injection procedure and setting. **eFigure 1.** Sensitivity analysis of EO rate in the office setting. **eFigure 2.** Overall rate of EO following intravitreal anti-VEGF injections in the OR setting by country development status. **eFigure 3.** Rate of EO following intravitreal ranibizumab injections in the OR setting by country development status. **eFigure 4.** Rate of EO following intravitreal bevacizumab injections in the OR setting by country development status. **eFigure 5.** Rate of culture-positive EO following intravitreal anti-VEGF injections in the OR setting by country development status. **eFigure 6.** Egger test results.
