# Supplementary material for: Safety of Receiving Anti–Vascular Endothelial Growth Factor Intravitreal Injection in Office-Based vs Operating Room Settings: A Meta-analysis
Source: JAMA Ophthalmol. 2021 Aug 19;139(10):1–10. doi: 10.1001/jamaophthalmol.2021.3096 (PMC8377608; doi:10.1001/jamaophthalmol.2021.3096)
Supplement: Supplement. — eMethods. Search strategies. eTable 1. Baseline characteristics of the included studies. eTable 2. Methodological qualities of the included studies. eTable 3. Characteristics of the injection procedure and setting. eFigure 1. Sensitivity analysis of EO rate in the office setting. eFigure 2. Overall rate of EO following intravitreal anti-VEGF injections in the OR setting by country development status. eFigure 3. Rate of EO following intravitreal ranibizumab injections in the OR setting by country development status. eFigure 4. Rate of EO following intravitreal bevacizumab injections in the OR setting by country development status. eFigure 5. Rate of culture-positive EO following intravitreal anti-VEGF injections in the OR setting by country development status. eFigure 6. Egger test results. [file jamaophthalmol-e213096-s001.pdf]

Supplemental Online Content

Li T, Sun J, Min J, et al. Safety of receiving anti–vascular endothelial growth factor intravitreal injection in office-based vs operating room settings: a meta-analysis. *JAMA Ophthalmol*. Published online August 19, 2021. doi:10.1001/jamaophthalmol.2021.3096

eMethods. Search strategies
eTable 1. Baseline characteristics of the included studies
eTable 2. Methodological qualities of the included studies
eTable 3. Characteristics of the injection procedure and setting
eFigure 1. Sensitivity analysis of EO rate in the office setting
eFigure 2. Overall rate of EO following intravitreal anti-VEGF injections in the OR setting by country development status
eFigure 3. Rate of EO following intravitreal ranibizumab injections in the OR setting by country development status
eFigure 4. Rate of EO following intravitreal bevacizumab injections in the OR setting by country development status
eFigure 5. Rate of culture-positive EO following intravitreal anti-VEGF injections in the OR setting by country development status
eFigure 6. Egger test results

This supplementary material has been provided by the authors to give readers additional information about their work.

© 2021 Li T et al. *JAMA Ophthalmology*.

eMethods. Search strategies

PubMed

(Lucentis or Ranibizumab or Eylea or Aflibercept or Conbercept or Beovu or Brolucizumab or bevacizumab or anti-VEGF) AND ("Operating Room" or "Office-Based" or Office or Operating Room[MeSH Terms]) and (“intravitreal injection” or IVI or intravitreal injection[MeSH Terms]) Sort by: Best Match Filters: English 49

1) Embase

(Lucentis OR Ranibizumab OR Eylea OR Aflibercept OR Conbercept OR Beovu OR Brolucizumab OR bevacizumab OR anti-VEGF) AND (‘Operating Room’ OR ‘Office-Based’ OR Office OR 'Operating Room'/exp) AND (‘intravitreal injection’ OR IVI OR ‘intravitreal injection’/exp) AND [embase]/lim AND [english]/lim 90

2) Cochrane Library

#1	(Lucentis or Ranibizumab or Eylea or Aflibercept or Conbercept or Beovu or Brolucizumab or bevacizumab or anti-VEGF)	8526
#2	("Operating Room" or "Office-Based" or Office)	17623
#3	MeSH descriptor: [Operating Room] explode all trees	212
#4	#2 or #3	15162
#5	(“intravitreal injection” or IVI)	2558
#6	MeSH descriptor: [Intravitreal Injections] explode all trees	721
#7	#5 or #6	1817
#8	#1 and #4 and #7	6

3) Web of Science

(Lucentis or Ranibizumab OR Eylea OR Aflibercept OR Conbercept OR Beovu OR Brolucizumab OR bevacizumab OR anti-VEGF) AND ("Operating Room" OR "Office-Based" OR office) AND (“intravitreal injection” OR IVI) Refined by: LANGUAGES: (ENGLISH) 37

4) ClinicalTrials.gov

Condition or disease: Intravitreal Injection

Study results: with results 80

© 2021 Li T et al. *JAMA Ophthalmology*.

eTable 1. Baseline characteristics of the included studies

First author, year	Study period, location	Injection setting	Anti-VEGF drug	Injection number	Patients (eyes)	Age (years)	Gender (F/M)	Dominant indication	Follow-up (M)	Study design	Safety outcomes
**Comparative studies**											
Tabandeh, 2014	2009 to 2011, USA and Italy	Office	Ranibizumab	2041	NR [a]	NR	NR	nAMD	NR	Retrospective, multicenter, case series	EO/Culture-positive EO
			Bevacizumab	6169							
			Total	8210							
		OR	Ranibizumab	683				nAMD	NR		
			Bevacizumab	2364							
			Total	3047							
Abell, 2012	2006 to 2012, Australia	Office	Ranibizumab, Bevacizumab	3376	1146 [b]	79.7±13.3	NR	/	NR	Retrospective, single-center, cohort study	EO/Culture-positive EO
		OR	Ranibizumab, Bevacizumab	8873		80.0±12.3	NR	/	NR		
**Single group studies - Office**											
Borkar, 2018	2012 to 2017, USA	Office	Bevacizumab	14339	5890	74.2±14.1	3571/2319	nAMD	12	Retrospective, single-center, case series	EO/RD
			Ranibizumab	55051							
			Aflibercept	32542							
			Total	101932							
Juncal, 2019	2016, Canada	Office	Ranibizumab	7824	262 (524)	76.8±11.1	151/111	nAMD	27.4±18.8 (1-76)	Retrospective, single-center, case series	EO/VH/RT/RD/ vascular-related systemic AEs
			Aflibercept	1860							
			Bevacizumab	114							
			Total	9798							
Goldberg, 2014	2011 to 2013, USA	Office	Aflibercept	5356	844	NR	NR	nAMD	NR	Retrospective, multicenter, case series	Noninfectious inflammation
Fintak, 2008	2005 to 2007, USA	Office	Bevacizumab	12585	NR	NR	NR	/	NR	Retrospective, multicenter,	EO/Culture-positive EO
			Ranibizumab	14320							

© 2021 Li T et al. *JAMA Ophthalmology*.

First author, year	Study period, location	Injection setting	Anti-VEGF drug	Injection number	Patients (eyes)	Age (years)	Gender (F/M)	Dominant indication	Follow-up (M)	Study design	Safety outcomes
	and Canada		Total	26905						case series	
Pilli, 2008	2005 to 2007, USA	Office	Bevacizumab	3501	NR	NR	NR	/	NR	Retrospective, single-center, case series	EO/Culture-positive EO
			Ranibizumab	6347							
			Total	9848							
Frenkel, 2010	NR, USA	Office	Bevacizumab	74	33	NR	NR	nAMD	6	Retrospective, single-center, case series	EO/RD/Cataracts/VH/RPE tear/myocardial infarction/stroke
			Ranibizumab	167	33						
			Total	241	59						
Haider, 2017	2015 to 2016, Pakistan	Office	Bevacizumab	1047	NR	NR	NR	NR	1	Prospective, single-center, case series	EO
Rayess, 2016	2009 to 2013, USA	Office	Bevacizumab	153812	NR	NR	NR	nAMD	NR	Retrospective, multicenter, cohort study	EO/Culture-positive EO
			Ranibizumab	309722							
			Aflibercept	40356							
			Total	503890							
Storey, 2013	2009 to 2012, USA	Office	Ranibizumab	71791	NR	NR	NR	NR	NR	Retrospective, single-center, case-control study	EO/Culture-positive EO
			Bevacizumab	44007							
			Aflibercept	1373							
			Total	117171							
Moshfeghi, 2011	2005 to 2010, USA	Office	Ranibizumab	18607	NR	NR	NR	NR	NR	Retrospective, single-center (multi-site), case series	EO/Culture-positive EO
			Bevacizumab	39700							
			Total	58307							
Chaudhary, 2013	2007 to 2011, USA	Office	Ranibizumab	20297	NR	NR	NR	NR	15 (5-48)	Retrospective, single-center, case series	EO/Culture-positive EO
			Bevacizumab	28705							
			Total	49002							
Shimada, 2013	2009 to	Office	Ranibizumab	13750	(2350)	NR	NR		At least 2	Retrospective,	EO

© 2021 Li T et al. *JAMA Ophthalmology*.

First author, year	Study period, location	Injection setting	Anti-VEGF drug	Injection number	Patients (eyes)	Age (years)	Gender (F/M)	Dominant indication	Follow-up (M)	Study design	Safety outcomes
	2012, Japan		Bevacizumab	846	(270)				months	single-center, case series	
			Total	14596	(2620)						
Cheung, 2012	2005 to 2010, Canada	Office	Ranibizumab	9453	/	/	/	Exudative AMD	NR	Retrospective, multi-center, case series	EO/Culture-positive EO
			Bevacizumab	5386							
			Total	14839							
Fineman, 2013	2010 to 2011, USA	Office	Ranibizumab	6330	NR	NR	NR	NR	NR	Retrospective, single-center, case series	EO/Culture-positive EO
			Bevacizumab	3834							
			Total	10164							
Englander, 2013	2007 to 2011, USA	Office	Ranibizumab	7768	/	NR	NR	nAMD		Retrospective, single-center, case series	EO/Culture-positive EO
			Bevacizumab	2315							
			Total	10083							
**Single group studies - OR**											
Jan, 2016	2008 to 2015, Pakistan	OR	Bevacizumab	6107	3570 (4352)	43 (5-81)	1334/2236	Diabetic macula oedema	20 days	Retrospective, single-center, case series	EO/RD
Freiberg, 2017	2003 to 2016, Switzerland and Denmark	OR	Bevacizumab, Ranibizumab, Aflibercept	134701	NR	NR	NR	NR	NR	Retrospective, multicenter, case series	EO/Culture-positive EO
Wani, 2016	2006 to 2012, Kuwait	OR	Bevacizumab	5429	NR	NR	NR	NR	NR	Retrospective, multicenter, case series	EO/Culture-positive EO/ RD
Hasler, 2015	2007 to 2011, Denmark	OR	Ranibizumab	38503	3679 (4623)	NR	NR	nAMD	12.2	Retrospective, single-center, case series	EO/Culture-positive EO/RD/Uveitis/ Cataracts/VH/ RPE tear
Casparis, 2014	2004 to	OR	Ranibizumab	36398	NR	NR	NR	NR	NR	Retrospective,	EO

© 2021 Li T et al. *JAMA Ophthalmology*.

First author, year	Study period, location	Injection setting	Anti-VEGF drug	Injection number	Patients (eyes)	Age (years)	Gender (F/M)	Dominant indication	Follow-up (M)	Study design	Safety outcomes
	2011, Switzerland		Bevacizumab	3518						multicenter, case series	
			Aflibercept	89							
			Total	40005							
Ou, 2019	2017, China	OR	Conbercept+ laser	NR	22 (22)	57.3±7.2	10/12	Retinal vein occlusion complicated with macular edema	6	Prospective, single-center, randomized controlled	EO/systemic reaction
			Ranibizumab+ laser		22 (22)	58.1±6.5	11/11				
			Total		44 (44)	/	21/23				
Zafar, 2018	2013 to 2015, Pakistan	OR	Bevacizumab	11128	1335	NR	NR	Diabetic retinopathy	NR	Retrospective, single-center, case series	EO/Culture-positive EO
			Ranibizumab		698						
			Aflibercept		21						
			Total		2054						
Zhang, 2015	2012 to 2013, China	OR	Ranibizumab	35	35 (35)	53.5 (26-69)	24/11	mCNV	20 (16-24)	Retrospective, single-center, case series	EO/Culture-positive EO/RD/uveitis/ thromboembolic event/systemic hypertension
Istek, 2014	2009 to 2012, Turkey	OR	Bevacizumab	NR	32	57.2 (34-78)	8/24	Macular edema secondary to branch retinal vein occlusion	12.37±0.73 (12-15)	Retrospective, single-center, case series	EO/systemic side effects

© 2021 Li T et al. *JAMA Ophthalmology*.

First author, year	Study period, location	Injection setting	Anti-VEGF drug	Injection number	Patients (eyes)	Age (years)	Gender (F/M)	Dominant indication	Follow-up (M)	Study design	Safety outcomes
Brynskov, 2014	2007 to 2013, Denmark	OR	Ranibizumab	20024	NR (2575)	NR	NR	NR	NR	Retrospective, single-center, case series	EO
			Aflibercept	269							
			Total	20293							
Mishra, 2018	2009 to 2016, India	OR	Ranibizumab	1832	13680	NR	NR	/	7 (1-78)	Retrospective, single-center, case series	EO/Culture-positive EO
			Bevacizumab	14124							
			Total	15956							
Nentwich, 2014	2005 to 2012, Germany	OR	Ranibizumab	10097	NR	NR	NR	NR	NR	Retrospective, single-center, case series	EO/Culture-positive EO
			Bevacizumab	7865							
			Total	17962							
**Single group studies – Office or OR**											
Busch, 2019	2011 to 2016 [c]	Office vs OR	Ranibizumab	NR	39	73±12	51/32 [d]	AMD	NR	Retrospective, multicenter, case series	EO/Culture-positive EO
			Aflibercept		22						
			Bevacizumab		17						
			Total		78						
Chen, 2011	2000 to 2010, USA	Office vs OR	Bevacizumab	6675	NR	NR	NR	EMD	NR	Retrospective, single-center, case series	EO/Culture-positive EO
			Ranibizumab	22336							
			Total	29011							

Data were expressed as mean±SD (range).

Note: AE: adverse event; EMD: exudative macular degeneration; F: female; M: male; mCNV: myopic choroidal neovascularization; nAMD: neovascular age-related macular degeneration; NR: not reported; OR: operating room; RD: retinal detachment; RPE: retinal pigment epithelial; RT: retinal tear; VH: vitreous hemorrhage; UK: United Kingdom; USA: United States of America; /: Relevant information had been reported, but could not extract data we needed.

a. There was no significant difference in rate of various baseline diagnosis and the type of medication that was injected.
b. There was no statistically significant difference in age, gender, smoking status, ocular comorbidities, or socioeconomic indexes between the two injection settings.
c. Multicenter study conducted in Germany, France, UK, Italy, Portugal, Spain, Turkey, Israel, Poland, Latvia, Australia, Thailand, and Argentina.
d. Among the 83 patients, 5 of them used unknown anti-VEGF drug.

© 2021 Li T et al. *JAMA Ophthalmology*.

eTable 2. Methodological qualities of the included studies

Randomized Controlled Trials (Cochrane handbook [a])						
First author, year	Domain 1	Domain 2	Domain 3	Domain 4	Domain 5	Overall risk of bias
	Randomization process	Deviations from the intended interventions	Missing outcome data	Measurement of the outcome	Selection of the reported result	
Ou, 2019	Some concerns	Low risk	Low risk	Low risk	Low risk	Some concerns

Case series (NICE [b]) (0=No, 1=Yes)								
First author, year	Case series collected in more than one center	Is the hypothesis/ aim/objective of the study clearly described	Are the inclusion and exclusion criteria (case definition) clearly reported	Is there a clear definition of the outcomes reported	Were data collected prospectively	Is there an explicit statement that patients were recruited consecutively	Are the main findings of the study clearly described	Are outcomes stratified
Busch, 2019	1	1	1	0	0	1	1	1
Jan, 2016	0	1	0	0	0	1	1	0
Borkar, 2018	0	1	1	1	0	1	1	0
Freiberg, 2017	1	1	1	1	0	1	1	0
Wani, 2016	1	1	1	1	0	1	1	0
Juncal, 2019	0	1	0	0	0	1	1	0
Tabandeh, 2014	1	1	0	1	0	1	1	0
Goldberg, 2014	1	1	0	1	0	1	1	0

© 2021 Li T et al. *JAMA Ophthalmology*.

Hasler, 2015	0	1	1	1	0	1	1	0
Fintak, 2008	1	1	0	1	0	1	1	0
Pilli, 2008	0	1	0	1	0	1	1	0
Casparis, 2014	1	1	0	1	1	1	1	0
Chen, 2011	0	1	1	1	0	1	1	1
Frenkel, 2010	0	1	0	1	0	0	1	0
Zafar, 2018	0	1	1	1	0	1	1	1
Haider, 2017	0	1	1	1	1	1	1	0
Zhang, 2015	0	1	1	1	0	1	1	0
Istek, 2014	0	1	1	1	0	1	1	0
Brynskov, 2014	0	1	1	1	0	0	1	0
Mishra, 2018	0	1	1	1	0	1	1	1
Moshfeghi, 2011	0	1	1	1	1	1	1	0
Chaudhary, 2013	0	1	1	1	1	1	1	0
Shimada, 2013	0	1	0	0	1	1	1	0
Cheung, 2012	1	1	1	1	1	1	1	1
Fineman, 2013	0	1	1	0	1	1	1	0
Englander,	0	1	1	1	1	1	1	0

© 2021 Li T et al. *JAMA Ophthalmology*.

2013								
Nentwich, 2014	0	1	1	1	1	1	1	0
**Cohort Studies (NOS [c])**								
First author, year	Selection				Comparability	Outcome		
	Representativeness of the exposed cohort	Selection of the non-exposed cohort	Ascertainment of exposure	Demonstration that outcome of interest was not present at start of study	Comparability of cohorts on the basis of the design or analysis	Assessment of outcome	Was follow-up long enough for outcomes to occur	Adequacy of follow up of cohorts
Abell, 2012	1	1	1	0	1	1	0	0
Rayess, 2016	1	1	1	1	0	1	1	0
**Case Control (NOS [c])**								
First author, year	Selection				Comparability	Exposure		
	Is the case definition adequate	Representativeness of the cases	Selection of controls	Definition of controls	Comparability of cases and controls on the basis of the design or analysis	Ascertainment of exposure	Same method of ascertainment for cases and controls	Non-Response rate
Storey, 2013	1	1	1	0	0	1	1	0

Note:

a. Cochrane handbook for systematic review of interventions. Available at: https://training.cochrane.org/handbook/current

b. NICE (National Institute for Health and Clinical Excellence): In one review published by NICE in 2005, the authors looked at the relationship between characteristics of case series studies and their outcomes. They found little evidence to support the use of many of the criteria included in tools used for quality assessment of case series studies. Reference: NICE Appendix 4 Quality of case series form [Z]. 2008: 2017.

c. The Newcastle-Ottawa Scale (NOS) is an ongoing collaboration between the Universities of Newcastle, Australia and Ottawa, Canada. It was developed to assess the quality of non-randomized studies with its design, content and ease of use directed to the task of incorporating the quality assessments in the interpretation of meta-analytic results. Available at: http://www.ohri.ca/programs/clinical_epidemiology/nosgen.pdf.

© 2021 Li T et al. *JAMA Ophthalmology*.

eTable 3. Characteristics of the injection procedure and setting

First author, year	Injection setting	Sterile condition (Y/N)									Same day bilateral injection	Drug preparation
		Gloves	Mask (propose silence)	Drape	Speculum	Hand antisepsis	Laminar airflow	PVI (Y/N)	Antibiotics (Y/N) Pre-injection	Antibiotics (Y/N) Post-injection		
**Comparative studies**												
Tabandeh, 2014	Office	N	N (N)	N	Y	NR	NR	Y	N	Y	NR	Bevacizumab was prepackaged by a compounding pharmacy. Ranibizumab was prepared before injection as per package instruction.
	OR	Y	Y (Y)	Y	Y	Y	NR	Y	Y	Y	NR	Bevacizumab was prepared from the stock bottle using a self-contained system for drug reconstitution, with a 0.22 µm filter. Bevacizumab and Ranibizumab were prepared before injection.
Abell, 2012	Office	Y	Y	Y	Y	NR	N	Y	NR	Y	NR	NR

© 2021 Li T et al. *JAMA Ophthalmology*.

First author, year	Injection setting	Sterile condition (Y/N)									Same day bilateral injection	Drug preparation
		Gloves	Mask (propose silence)	Drape	Speculum	Hand antisepsis	Laminar airflow	PVI (Y/N)	Antibiotics (Y/N)			
									Pre-injection	Post-injection		
	OR	Y	Y	Y	Y	NR	NR	Y	NR	N	NR	
**Single group studies - Office**												
Borkar, 2018	Office	NR	NR	NR	NR	NR	NR	Y	NR	Y	Y	Bevacizumab injections were administered using prepackaged syringes. Aflibercept syringes were loaded from single-use vials. Ranibizumab injections were administered using either syringes loaded from single-use vials or prefilled syringes.
Juncal, 2019	Office	N	N (Y)	NR	Y	NR	NR	Y	N	N	Y	Aflibercept (2.0 mg/0.05 mL) syringes were withdrawn from single-use vials. Bevacizumab (1.25 mg/0.05 mL) injections consisted of prepackaged

© 2021 Li T et al. *JAMA Ophthalmology*.

First author, year	Injection setting	Sterile condition (Y/N)									Same day bilateral injection	Drug preparation
		Gloves	Mask (propose silence)	Drape	Speculum	Hand antisepsis	Laminar airflow	PVI (Y/N)	Antibiotics (Y/N)			
									Pre-injection	Post-injection		
												syringes. Ranibizumab (0.5 mg/0.05 mL) injections included both prefilled syringes or were loaded from single-use vials.
Goldberg, 2014	Office	NR	NR	NR	NR	NR	NR	Y	NR	Y	Y [a]	Aflibercept are delivered in single dose, sterile glass vials.
Fintak, 2008	Office	NR	NR	NR	TBD [b]	NR	NR	Y	NR	Y	NR	Most intravitreal injections of Bevacizumab given were aliquoted into a 1 mL syringe by a compounding pharmacy. Bevacizumab (1.25 mg/ 0.05 mL) was in multiuse vial and ranibizumab (0.5 mg/ 0.05 mL) in single use vial.
Pilli, 2008	Office	NR	NR	N	TBD [b]	NR	NR	Y	N	NR	NR	NR

© 2021 Li T et al. *JAMA Ophthalmology*.

First author, year	Injection setting	Sterile condition (Y/N)									Same day bilateral injection	Drug preparation
		Gloves	Mask (propose silence)	Drape	Speculum	Hand antisepsis	Laminar airflow	PVI (Y/N)	Antibiotics (Y/N)			
									Pre-injection	Post-injection		
Frenkel, 2010	Office	N [c]	NR	NR	TBD [b]	NR	NR	Y	Y	Y	NR	NR
Haider, 2017	Office	Y	Y	Y	Y	Y	NR	Y	Y	Y	NR	A registered pharmacist formulated the injection preparation of bevacizumab and they were then delivered to the hospital.
Rayess, 2016	Office	NR	NR	NR	Y	NR	NR	Y	N	Y [d]	NR	Bevacizumab was repackaged into syringes at compounding pharmacies; ranibizumab and aflibercept syringes were loaded from single use vials.
Storey, 2013	Office	N	N	N	TBD [e]	NR	NR	Y	Y	Y [f]	NR	NR
Moshfeghi, 2011	Office	N [e]	N	N	Y	NR	NR	Y	N	Y [g]	NR	NR

© 2021 Li T et al. *JAMA Ophthalmology*.

First author, year	Injection setting	Sterile condition (Y/N)									Same day bilateral injection	Drug preparation
		Gloves	Mask (propose silence)	Drape	Speculum	Hand antisepsis	Laminar airflow	PVI (Y/N)	Antibiotics (Y/N)			
									Pre-injection	Post-injection		
Chaudhary, 2013	Office	N [h]	N	N	Y	NR	NR	Y	Y	Y	NR	NR
Shimada, 2013	Office	Y	Y (Y)	Y	Y	NR	NR	Y	Y	Y	NR	NR
Cheung, 2012	Office	Y [i]	N	Y [i]	Y	NR	NR	Y	Y [i]	Y [i]	NR	NR
Fineman, 2013	Office	N	N	N	N	NR	NR	Y	N	Y [j]	NR	NR
Englander, 2013	Office	Y [k]	Y [k]	Y [k]	Y [k]	NR	NR	Y	Y [k]	Y [k]	NR	NR
**Single group studies - OR**												
Jan, 2016	OR	Y	NR	NR	Y	NR	NR	Y	NR	Y	NR	Bevacizumab injections were prepared from same vial by multiple withdrawals taking care of aseptic precautions. Bevacizumab 0.05 ml was taken aseptically from the vial into 30G insulin

© 2021 Li T et al. *JAMA Ophthalmology*.

First author, year	Injection setting	Sterile condition (Y/N)									Same day bilateral injection	Drug preparation
		Gloves	Mask (propose silence)	Drape	Speculum	Hand antisepsis	Laminar airflow	PVI (Y/N)	Antibiotics (Y/N)			
									Pre-injection	Post-injection		
												syringe and was injected infero-temporally.
Freiberg, 2017	OR	Y	Y	Y	Y	NR	Y	Y	N	Y [l]	NR	Bevacizumab (1.25 mg/0.05 mL) was prepared aseptically for intraocular use by licensed and certified compounding pharmacies. Ranibizumab came in prefilled syringes. Aflibercept were supplied by the relevant manufacturer.
Wani, 2016	OR	Y	Y	Y/N [m]	Y/N [n]	Y	N	Y	NR	Y	NR	All 4 ml of bevacizumab are withdrawn with a sterile 5 ml syringe. The needle is removed and a new needle is fixed to the 5 ml syringe and 0.1 ml of bevacizumab are

© 2021 Li T et al. *JAMA Ophthalmology*.

First author, year	Injection setting	Sterile condition (Y/N)									Same day bilateral injection	Drug preparation
		Gloves	Mask (propose silence)	Drape	Speculum	Hand antisepsis	Laminar airflow	PVI (Y/N)	Antibiotics (Y/N)			
									Pre-injection	Post-injection		
												transferred to the insulin syringes with the piston removed by the scrub nurse. The piston of the insulin syringe is replaced and the dose is adjusted to 0.05 ml. This procedure is repeated until the required number of syringes are loaded with bevacizumab.
Hasler, 2015	OR	Y	Y	Y	Y	NR	NR	Y	N	Y	NR	Ranibizumab (0.05 ml) was delivered in custom packages including syringes, needles and medicine.
Casparis, 2014	OR	Y	Y	Y	Y	NR	Y	Y	N	Y/N °	NR	Commercially available Bevacizumab was prepared aseptically for intraocular use (1.25

© 2021 Li T et al. *JAMA Ophthalmology*.

First author, year	Injection setting	Sterile condition (Y/N)									Same day bilateral injection	Drug preparation
		Gloves	Mask (propose silence)	Drape	Speculum	Hand antisepsis	Laminar airflow	PVI (Y/N)	Antibiotics (Y/N)			
									Pre-injection	Post-injection		
												mg/0.05 mL) by local certified compounding pharmacies. Ranibizumab and Aflibercept were used as supplied by the relevant manufacturer.
Ou, 2019	OR	NR	NR	NR	NR	NR	Y	NR	NR	Y	N	NR
Zafar, 2018	OR	Y	Y (Y)	Y	Y	Y	NR	Y	N	Y	NR	NR
Zhang, 2015	OR	NR	NR	NR	Y [b]	NR	NR	Y	NR	NR	N	NR
Istek, 2014	OR	NR	NR	NR	NR	NR	NR	Y	NR	NR	NR	NR
Brynskov, 2014	OR	Y	Y	Y	Y	Y	NR	Y	N	Y	N	NR
Mishra, 2018	OR	Y	Y	Y	Y [b]	NR	NR	Y	Y	Y	NR	The dosages administered were 1.25 mg (0.05 mL of 25 mg/mL) of bevacizumab, 0.5 mg (0.05 mL of 10 mg/mL) of ranibizumab. From 2009

© 2021 Li T et al. *JAMA Ophthalmology*.

First author, year	Injection setting	Sterile condition (Y/N)									Same day bilateral injection	Drug preparation
		Gloves	Mask (propose silence)	Drape	Speculum	Hand antisepsis	Laminar airflow	PVI (Y/N)	Antibiotics (Y/N)			
									Pre-injection	Post-injection		
												to 2011, the Bevacizumab injections were drawn from a single vial. From 2012-2016, bevacizumab was dispensed aseptically in an in-house compounding pharmacy from a single 4 mL vial into 25 single 1cc tuberculin syringes and individually packaged.
Nentwich, 2014	OR	Y	Y	Y	Y	NR	NR	Y	N	Y	NR	NR
**Single group studies – Office or OR**												
Busch, 2019	Office vs OR	Y [i]	Y [i]	NR	NR	NR	NR	NR	Y [i]	Y [i]	NR	NR
Chen, 2011	Office vs OR	NR	NR	NR	NR	NR	NR	Y	Y	Y	NR	NR

Note: OR: operating room; NR: not reported;

a. Four patients received bilateral injections with aflibercept.
b. There is no description in the study that whether the speculum is sterile or not.
c. Nonsterile gloves were used.
d. During the initial period of the study, patients were routinely prescribed post-injection topical antibiotics for prophylaxis, followed by a transition period in which some physicians continued. Finally, antibiotics were not

© 2021 Li T et al. *JAMA Ophthalmology*.

routinely prescribed.

e. Physicians individually determined use of a bladed lid speculum.
f. All patients from January 1, 2009, through April 30, 2011, received post-injection antibiotics, whereas all patients from January 1, 2012, through October 1, 2012, did not receive.
g. According to physician preference.
h. Some physicians wore nonsterile gloves whereas some wore no gloves at all.
i. Part of the patients used.
j. Used until Oct 2011.
k. Part of the treating ophthalmologists used.
l. Postoperative antibiotics varied among sites.
m. Drape was used only in one site.
n. Speculum was used only in one site.
o. Post-injection topical antibiotics were used only in one hospital.

© 2021 Li T et al. *JAMA Ophthalmology*.

eFigure 1. Sensitivity analysis of EO rate in the office setting

```
Influential analysis (Fixed effect model)

                          events           95%-CI p-value   tau^2    tau     I^2
Omitting Tabandeh 2014    0.0336 [0.0301; 0.0375]          0.0082  0.0907  10.4%
Omitting Abell 2012       0.0333 [0.0298; 0.0372]          0.0101  0.1003  12.3%
Omitting Borkar 2018      0.0344 [0.0306; 0.0386]          0.0000  0.0000   0.0%
Omitting Juncal 2019      0.0339 [0.0303; 0.0378]          0.0016  0.0395   2.2%
Omitting Fintak 2008      0.0340 [0.0304; 0.0380]          0.0000  0.0000   0.0%
Omitting Pilli 2008       0.0337 [0.0301; 0.0376]          0.0073  0.0855   9.3%
Omitting Frenkel 2020     0.0336 [0.0301; 0.0376]          0.0066  0.0812   8.6%
Omitting Haider 2017      0.0335 [0.0300; 0.0374]          0.0092  0.0961  11.6%
Omitting Rayess 2016      0.0305 [0.0258; 0.0362]          0.0129  0.1136   9.2%
Omitting Storey 2013      0.0331 [0.0294; 0.0373]          0.0184  0.1358  16.1%
Omitting Moshfeghi 2011   0.0345 [0.0308; 0.0386]          0.0000  0.0000   0.0%
Omitting Chaudhary 2013   0.0336 [0.0300; 0.0376]          0.0150  0.1223  16.0%
Omitting Shimada 2013     0.0342 [0.0306; 0.0382]          0.0000  0.0000   0.0%
Omitting Cheung 2012      0.0334 [0.0299; 0.0374]          0.0112  0.1057  13.2%
Omitting Fineman 2013     0.0337 [0.0302; 0.0376]          0.0071  0.0843   9.1%
Omitting Englander 2013   0.0337 [0.0301; 0.0376]          0.0072  0.0846   9.1%

Pooled estimate           0.0336 [0.0301; 0.0376]          0.0067  0.0820   8.3%

Details on meta-analytical method:GLMM
- Maximum-likelihood estimator for tau^2
- Logit transformation
```

© 2021 Li T et al. *JAMA Ophthalmology*.

eFigure 2. Overall rate of EO following intravitreal anti-VEGF injections in the OR setting by country development status

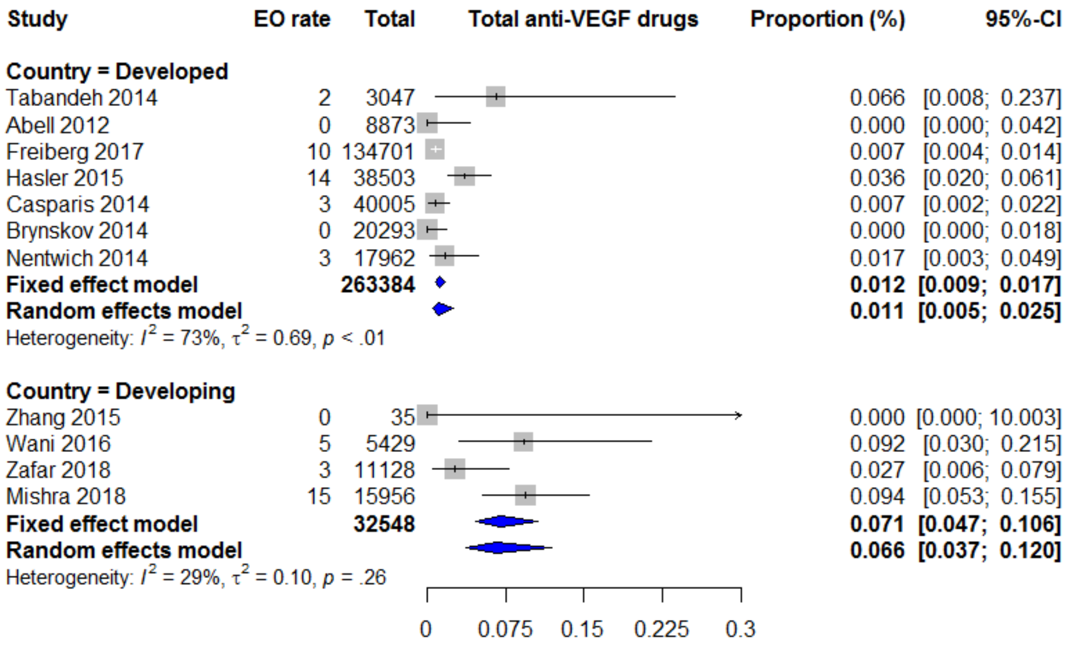

© 2021 Li T et al. *JAMA Ophthalmology*.

eFigure 3. Rate of EO following intravitreal ranibizumab injections in the OR setting by country development status

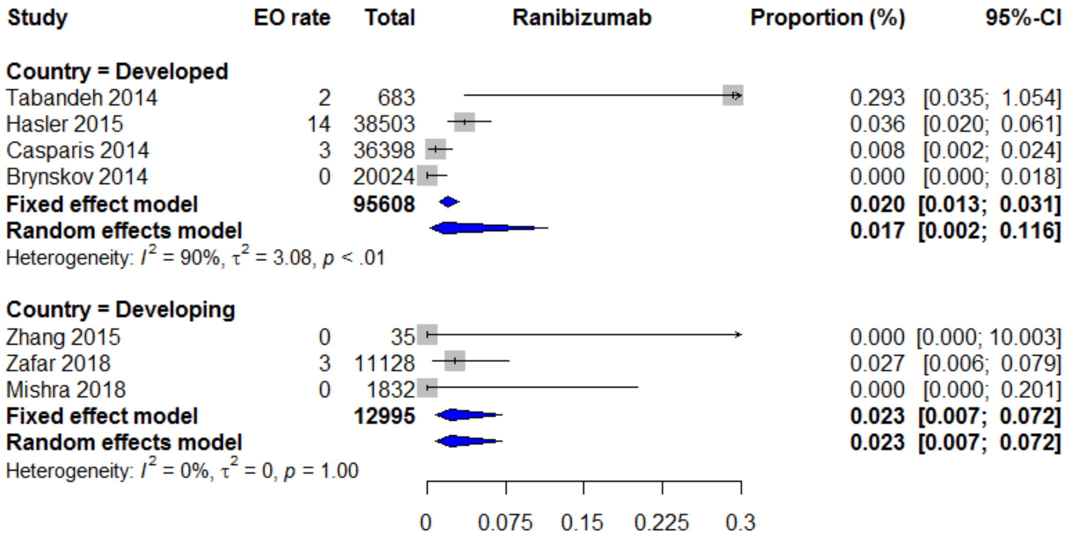

© 2021 Li T et al. *JAMA Ophthalmology*.

eFigure 4. Rate of EO following intravitreal bevacizumab injections in the OR setting by country development status

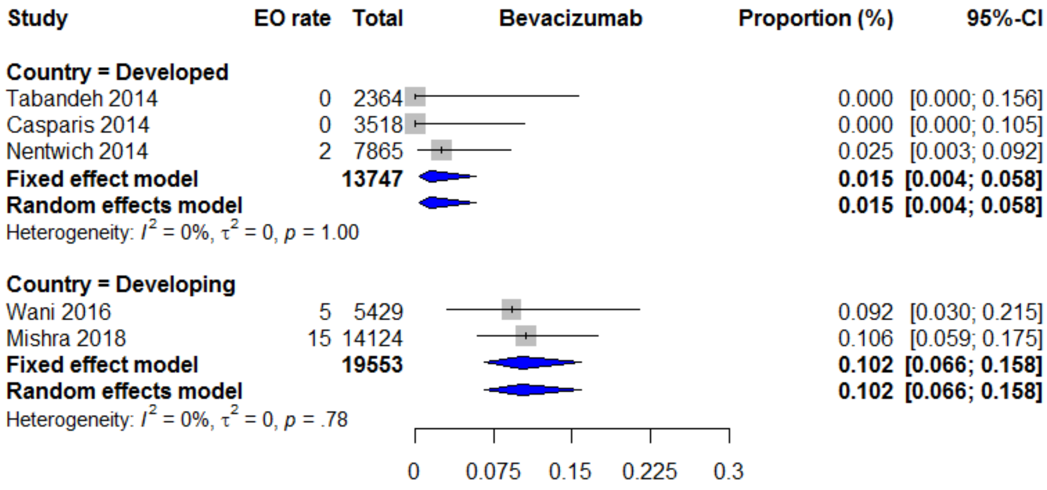

© 2021 Li T et al. *JAMA Ophthalmology*.

eFigure 5. Rate of culture-positive EO following intravitreal anti-VEGF injections in the OR setting by country development status

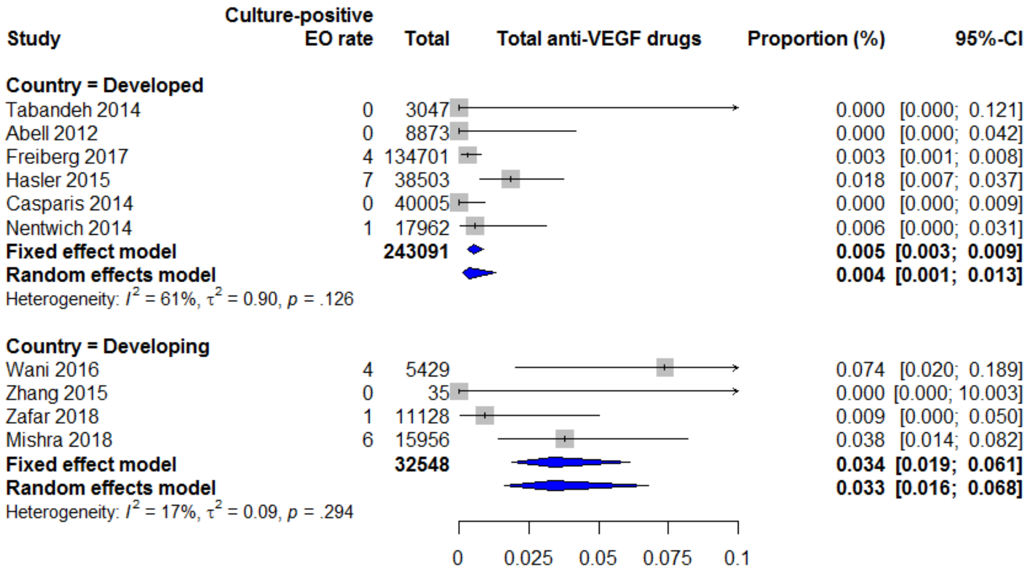

eFigure 6. Egger test results

A. EO rate in the office setting of total anti-VEGF drugs

```
        Linear regression test of funnel plot asymmetry

data:  metarate
t = -0.068492, df = 14, p-value = 0.9464
alternative hypothesis: asymmetry in funnel plot
sample estimates:
       bias     se.bias   intercept
-0.03274852  0.47813536 -7.94752291
```

B. EO rate in the office setting of ranibizumab

```
        Linear regression test of funnel plot asymmetry

data:  metaoffra
t = -1.6397, df = 12, p-value = 0.127
alternative hypothesis: asymmetry in funnel plot
sample estimates:
      bias    se.bias  intercept
-0.5951780  0.3629725 -7.9236064
```

C. EO rate in the office setting of bevacizumab

```
        Linear regression test of funnel plot asymmetry

data:  metaoffbe
t = 1.1396, df = 13, p-value = 0.275
alternative hypothesis: asymmetry in funnel plot
sample estimates:
      bias    se.bias  intercept
 0.5471218  0.4800937 -7.9293422
```

D. EO rate in the operating room of total anti-VEGF drugs

```
        Linear regression test of funnel plot asymmetry

data:  metarateor
t = -0.475, df = 9, p-value = 0.6461
alternative hypothesis: asymmetry in funnel plot
sample estimates:
      bias    se.bias  intercept
-0.7769404  1.6356736 -7.6955104
```

E. Culture-positive EO rate in the office setting of total anti-VEGF drugs

© 2021 Li T et al. *JAMA Ophthalmology*.

```
        Linear regression test of funnel plot asymmetry

data:  metacurate
t = 0.94536, df = 8, p-value = 0.3722
alternative hypothesis: asymmetry in funnel plot
sample estimates:
      bias    se.bias  intercept
 0.5363138  0.5673139 -8.9338570
```

F. Culture-positive EO rate in the operating room of total anti-VEGF drugs

```
        Linear regression test of funnel plot asymmetry

data:  metacuor
t = -0.36993, df = 8, p-value = 0.721
alternative hypothesis: asymmetry in funnel plot
sample estimates:
      bias    se.bias  intercept
-0.5442677  1.4712798 -8.2697033
```

© 2021 Li T et al. *JAMA Ophthalmology*.
